# Online Communication Attitudes and Video Game Co-Play in Older Adults: Cross-Sectional Mediation Study

**DOI:** 10.2196/80541

**Published:** 2026-04-07

**Authors:** Jeffrey Tsifan Tseng

**Affiliations:** 1 Department of Communication, University of California, Davis, 1 Shields Ave, Davis, CA, 95616, United States, 1 3179031603

**Keywords:** older adults, video game co-play, digital health, social support, intergenerational communication, online communication attitudes, uses and gratifications theory

## Abstract

**Background:**

Social isolation among older adults is a growing public health concern. While information and communication technologies offer opportunities for social engagement, few studies have examined how video game co-play, a form of interactive digital media, supports intergenerational connection and perceived social support among older adults.

**Objective:**

Guided by uses and gratifications theory, this study investigates whether older adults’ online communication attitudes predict video game co-play with younger family members and whether such co-play enhances perceived social support.

**Methods:**

A total of 433 older adults (aged ≥60 years) were recruited using a snowball sampling approach facilitated by undergraduate students. Participants completed an online survey assessing online social connection; online self-disclosure (OSD); video game co-play with younger family members; and perceived informational, instrumental, and emotional support. Regression analyses predicting co-play behavior (H1) were conducted using the full sample (N=433), with participants reporting no co-play coded as 0 to indicate the absence of engagement. Hierarchical regression analyses examining associations between co-play and social support (H2) were restricted to participants reporting video game co-play (n=71). Mediation analyses testing indirect effects (H3-H4) were also conducted among co-players (n=71), controlling for age, gender, and race. Mediation models were estimated using PROCESS Model 4, with 5000 bootstrap samples to generate 95% CIs.

**Results:**

OSD was positively associated with video game co-play behavior (*b*=0.12, SE=0.03; *P*<.001), whereas online social connection was not significantly related to co-play (*b*=0.06, SE=0.03; *P*=.08). Video game co-play was positively associated with perceived informational (*b*=0.11, SE=0.04; *P*=.01) and instrumental (*b*=0.16, SE=0.04; *P*<.001) social support but not emotional support (*b*=0.07, SE=0.04; *P*=.053). Among 71 participants reporting co-play, mediation analyses indicated that video game co-play mediated the association between OSD and informational support (indirect effect *b*=0.01, 95% CI .001-0.03) and instrumental support (indirect effect *b*=0.02, 95% CI 0.005-0.03), with partial mediation for informational support and full mediation for instrumental support.

**Conclusions:**

While not interventional, these findings suggest that video game co-play may function as a culturally relevant, digitally mediated relational practice through which older adults engage in intergenerational connection and perceive informational and instrumental support. The results inform digital health efforts aimed at reducing social isolation and underscore the importance of integrating digital literacy and attitudinal readiness into age-inclusive policy and program design. However, the small subsample of older adults who reported co-play behavior warrants caution in generalizing the findings and highlights the need for broader inclusion in future research.

## Introduction

### Background

As the global population ages, addressing social isolation among older adults has become a pressing public health concern. Digital health tools, especially information and communication technologies (ICTs), have emerged as promising means to support emotional well-being and social connectivity [1,2]. Among these tools, video games are increasingly recognized not just as recreational platforms but as interactive environments capable of facilitating intergenerational engagement [3]. As digital connectivity among older adults continues to grow, there is a critical need to examine the psychological drivers that shape their engagement in online activities and evaluate the social and relational benefits that follow [4,5].

Older adults’ online communication attitudes, specifically online self-disclosure (OSD) and online social connection (OSC), may influence how they interact with digital platforms [6,7]. OSD refers to the willingness to share personal thoughts and experiences online, while OSC reflects the perception that digital tools help sustain interpersonal bonds [7,8]. These attitudes influence participation in relationship-enhancing digital activities, such as video game co-play.

Video game co-play, which involves collaborative or competitive gaming between older adults and younger family members, enables shared interactive experiences that extend beyond entertainment [3]. Through gameplay, participants engage in storytelling, problem-solving, and coordinated activity, processes that have been shown to strengthen family closeness, shared identity, and intergenerational understanding [9-11]. Prior research also suggests that co-play can foster emotional support and a sense of inclusion in digital spaces, particularly for older adults navigating geographical distance from family members [12,13].

Unlike more passive or asynchronous ICTs, such as text messaging or social media browsing, video game co-play is characterized by real-time interaction, shared goals, and sustained mutual attention [3,11,14]. These affordances create structured opportunities for collaboration, reciprocity, and informal support exchange, making co-play particularly well suited for intergenerational connection [3,11,12]. Focusing on co-play allows this study to isolate a form of digital engagement that is both socially immersive and relationally consequential for older adults [9,12].

To explore these dynamics, this study draws on uses and gratifications theory (UGT) as a foundational framework for understanding older adults’ agency in selecting and engaging with digital media [15,16]. While UGT has traditionally been applied to explain media choice and individual-level gratifications, recent scholarship highlights the need to extend the theory to interactive, relational, and socially embedded digital practices [16], particularly in aging populations [2,17]. Within this expanded perspective, older adults’ online communication attitudes, such as openness to self-disclosure and perceived importance of online connection, may shape their engagement in interactive activities, such as video game co-play, which offer opportunities for synchronous communication and shared relational experiences across generations.

Social support, including informational, instrumental, and emotional forms, is a key determinant of psychological well-being in later life [18,19]. In digital intergenerational contexts, support may emerge through cooperative gameplay and emotionally resonant exchanges [20,21]. Understanding these relationships provides insight into how digital tools can sustain social bonds and promote resilience among older adults.

By conceptualizing video game co-play as a digitally mediated relational practice rather than a formal intervention, this study highlights a culturally situated pathway through which older adults may sustain intergenerational connection across geographic distance. In doing so, it extends UGT to later life by emphasizing how motivational dispositions translate into interactive relational behaviors, while also acknowledging that access, digital literacy, and cultural norms may shape engagement. These insights provide a theoretically grounded foundation for future research and age-inclusive digital engagement strategies.

### Literature Review: Uses and Gratifications Theory

UGT provides a foundational framework for understanding how individuals actively select media to fulfill specific psychological and social needs [15]. Unlike passive models of media effects, UGT emphasizes users’ agency in engaging with communication technologies based on anticipated gratifications such as entertainment, escapism, information-seeking, and social connection [22,23]. Although originally developed in the context of mass media, contemporary extensions of UGT emphasize its applicability to interactive, socially embedded, and relational digital practices, moving beyond individual media choice to account for co-use and participatory engagement [16,23]. This approach underscores the functional role of media in addressing users’ everyday emotional and relational goals.

For older adults, UGT provides valuable insights into the adoption of digital tools designed to enhance well-being and maintain social relationships. Research has shown that motivations, such as companionship, leisure, and family connection, are central drivers of technology use in aging populations [2,16]. These motivations are especially salient in intergenerational contexts, where digitally mediated shared activities, such as video game co-play, can foster emotional reciprocity and familial closeness.

UGT also emphasizes the significance of individual differences, particularly in attitudes toward digital communication, in determining how and why technologies are utilized. Individuals actively choose media based on psychological and social needs, and these choices vary according to personal dispositions, such as motivation, past experiences, and social context [23]. For older adults, attitudinal orientations, such as openness to self-disclosure or valuing online social connection, may influence not only whether they engage with specific digital platforms but also how effectively those tools meet their evolving social needs. Understanding these motivations is crucial for informing age-appropriate digital engagement strategies that extend beyond usability to foster lasting and meaningful participation. As such, UGT provides a valuable framework for examining how older adults integrate video game co-play into their relational routines, particularly as a means of maintaining connections and reducing isolation in long-distance family relationships. Building on this perspective, this study advances UGT by applying it to intergenerational digital interaction in later life, emphasizing how attitudinal orientations toward online communication shape engagement in interactive, relational media practices rather than passive consumption.

### Significance of Video Game Co-Play for Older Adults

Video game co-play has emerged as an innovative and increasingly meaningful form of intergenerational interaction, particularly for older adults seeking to sustain connections with younger family members [3,12]. More than a source of entertainment, co-playing video games provides a structured, immersive experience that fosters real-time engagement, reciprocal communication, and collaborative problem-solving [3,14]. These interactions can serve as digital pathways for maintaining familial bonds and promoting intergenerational understanding.

For older adults, video game co-play can yield significant emotional and psychosocial benefits. Shared gameplay fosters mutual support and relational closeness, buffering against the health risks of isolation [14,24]. Studies found that older adults who engaged in digital gaming reported significantly higher levels of emotional well-being and reduced depressive symptoms compared to nonplayers [24]. Moreover, social gaming interventions have demonstrated feasibility and effectiveness in promoting intergenerational connection and alleviating loneliness. For example, past study has found that mobile gaming apps designed to facilitate intergenerational interaction can help to increase engagement and reduce social isolation among older participants [25]. These experiences enhance inclusion and self-worth, improving emotional health in later life.

Importantly, video game co-play supports synchronous engagement across physical distances, making it especially relevant for families separated by geography or mobility constraints [12,26]. Unlike more passive forms of communication, such as text messaging or phone calls, gameplay fosters dynamic interaction and shared accomplishment. Through these playful yet meaningful exchanges, older adults and younger relatives can maintain continuity in their relationships despite limited face-to-face contact [27].

In this sense, video game co-play can be understood as a low-barrier, home-based digitally mediated relational practice that holds relevance for digital health and social inclusion efforts. Its accessibility, emotional salience, and interactive format make it a promising tool for enhancing relational satisfaction and psychological well-being among older populations. As such, integrating co-play into broader digital health strategies and aging support programs may offer new avenues to promote social connectedness in aging communities.

Importantly, cross-cultural and comparative research suggests that engagement in intergenerational digital activities is deeply shaped by sociocultural norms related to family obligation, leisure, and emotional expression [28,29]. Prior research indicates that collectivist cultural contexts may place greater emphasis on shared activities and instrumental caregiving, potentially influencing both the adoption of intergenerational co-play and perceptions of its relational value [30,31]. In such contexts, shared digital practices may function as extensions of everyday family caregiving rather than as purely recreational activities [32]. Recognizing these cultural contingencies is essential for interpreting findings related to intergenerational digital engagement and for avoiding overgeneralization across diverse aging populations.

### Online Communication Attitudes

Within the UGT framework, online communication attitudes serve as important predictors of media selection and engagement. Drawing on the social influence model of technology use, research shows that individuals’ engagement with digital platforms is shaped by their preexisting attitudes toward mediated communication [33,34]. This highlights the central role of attitudinal dispositions, particularly among older adults, in influencing how and why digital tools are used. Attitudes are composed of both affective and cognitive dimensions, which guide individuals’ behavioral tendencies [35]. The theory of reasoned action further posits that attitudes and behavioral intentions are key predictors of actual behavior, particularly in contexts that are socially salient, such as maintaining family relationships [36].

Two primary components of online communication attitudes include OSC and OSD [6,8]. OSC refers to the belief that digital technologies are essential for sustaining interpersonal relationships, while OSD reflects one’s comfort with sharing personal information in online environments [6,7]. Individuals high in OSC may view digital engagement as a necessary aspect of their social well-being, whereas those high in OSD tend to interact more frequently and meaningfully across digital platforms [6-8]. These dimensions are especially relevant in older populations, as they help explain variability in how digital tools are adopted and used for relationship maintenance.

In the context of aging and family communication, OSC holds particular significance. For older adults with mobility limitations or who are geographically separated from loved ones, maintaining close contact often relies on digital channels [1,37]. Technologies, such as video games, offer new, interactive avenues for sustaining these relationships. Prior studies have shown that older adults who view online platforms as socially vital are more inclined to adopt tools that promote interactive engagement, including video game co-play [2,38]. From this perspective, co-play can be seen as a relational strategy grounded in OSC, a form of active, synchronous interaction that reinforces intergenerational connection.

Similarly, OSD may be a critical enabler of co-play behavior. The willingness to share personal experiences and emotions in digital contexts can deepen relationships and foster more meaningful interactions [7]. In video game environments, where gameplay often involves storytelling, collaboration, and real-time feedback [3], older adults who are comfortable with online self-disclosure may be more inclined to engage. Research has found that individuals high in OSD tend to be more active across various forms of digital media, including those that support emotionally rich exchanges [39,40]. This openness may enhance the depth of intergenerational connection within the gaming context.

Building on these insights, this study predicts that online communication attitudes, specifically OSC and OSD, will significantly influence the extent to which older adults participate in video game co-play with younger family members.

Hypothesis 1 (H1): (a) OSC and (b) OSD will be significantly associated with video game co-play behavior among older adults in the context of long-distance family relationships.

### Video Game Co-Play and Perceived Social Support

Building on the understanding that attitudes influence technology adoption and use [39,41,42], the construct of perceived social support is central to evaluating how digital interaction fosters relational well-being among older adults. Perceived social support encompasses several key dimensions: informational support (eg, sharing advice and guidance), instrumental or tangible support (eg, providing assistance or resources), emotional support (eg, offering empathy and care), social network support (eg, fostering connectedness and belonging), and esteem support (eg, affirming personal worth) [43]. These forms of support collectively contribute to emotional resilience and psychological health in later life.

In intergenerational contexts, family members are typically the primary providers of social support. Emotional support from close relatives is strongly associated with increased life satisfaction and psychological well-being in older adults [44], while instrumental support is essential for navigating health care systems, managing daily tasks, and responding to crises [45]. Informational support, in turn, plays a critical role in promoting autonomy and informed decision-making [46]. These resources support older adults’ well-being and integration [47].

When geographic distance limits in-person interaction, digital communication technologies become vital tools for sustaining these support systems. Research indicates that ICTs enable older adults to maintain emotional intimacy and receive both practical and emotional assistance across distances [48,49]. Through interactive platforms, such as video games, older adults can share experiences, collaborate on tasks, and engage in emotionally resonant dialog with younger family members [38,50]. These forms of digital engagement can reinforce a sense of belonging, strengthen emotional ties, and affirm older adults’ roles within their family networks.

Empirical studies have documented how ICT engagement improves perceptions of social support and reduces feelings of loneliness [17,51]. In senior living settings, technology use has been linked to enhanced social interaction, community integration, and emotional well-being [1]. Applying these findings to video game co-play, it is reasonable to hypothesize that engaging in shared digital gameplay with younger family members may lead to increased perceptions of informational, instrumental, and emotional support, especially in the context of long-distance relationships.

Hypothesis 2 (H2): Video game co-play will be positively associated with perceived (a) informational, (b) instrumental, and (c) emotional social support among older adults in long-distance relationships.

### Mediating Role of Video Game Co-Play

While general ICT use has been shown to mediate the relationship between online communication attitudes and perceived social support [52], video game co-play represents a particularly interactive and emotionally rich form of digital engagement that may offer a stronger pathway to familial bonding [14,27]. Older adults who exhibit positive attitudes toward OSC and OSD are more likely to adopt collaborative digital platforms that enable synchronous, emotionally meaningful interaction [2,7]. Video game co-play exemplifies such a platform, providing not only entertainment but also opportunities for shared achievement, relational expression, and emotional reciprocity [3].

Co-playing video games encourages sustained communication, cooperation, and joint problem-solving, which are core components of emotionally supportive relationships and intergenerational understanding [3,27]. These dynamics are especially salient for older adults seeking to maintain connections with geographically distant family members. The interactive nature of co-play allows older users to both express care and receive encouragement through real-time engagement, reinforcing feelings of value and inclusion within family networks [17]. This process not only enhances emotional closeness but also strengthens perceptions of informational, instrumental, and emotional support [38,51].

Based on this theoretical and empirical foundation, this study examines a mediation model in which video game co-play serves as the mechanism through which online communication attitudes influence perceived social support among older adults. This framework positions co-play not merely as an outcome of digital attitudes but as a critical conduit for transforming psychological dispositions into relational benefits in digitally mediated family interactions.

Hypothesis 3 (H3): Video game co-play will mediate the relationship between OSC and perceived (a) informational, (b) instrumental, and (c) emotional social support among older adults in long-distance family relationships.

Hypothesis 4 (H4): Video game co-play will mediate the relationship between OSD and perceived (a) informational, (b) instrumental, and (c) emotional social support among older adults in long-distance family relationships.

## Methods

### Participants

Older adults aged 60 years and above were recruited using a snowball sampling approach through undergraduate students at a large West Coast university. Each college-aged participant was instructed to invite 1 older adult family member (aged 60 years or older) who resided in a different geographic location. The long-distance nature of the intergenerational dyads was determined based on participant self-report and supplemented by IP address filtering to reduce the likelihood that dyad members were responding from the same physical location. This recruitment method was incentivized through extra credit for the undergraduate students; no monetary or nonmonetary incentives were provided to older adult participants.

Initially, a total of 1306 older adult participants were recruited (mean age 70.14, SD 7.23 y). To ensure data quality, participants who had excessive missing data or spent insufficient time on the survey (ie, less than 300 s, based on pilot test standards) were excluded, resulting in the removal of 638 cases. An additional 235 participants were excluded due to missing data on the video game co-play measure, defined as incomplete or nonresponse to the co-play items administered to participants who indicated engagement in video game play. The final sample consisted of 433 older adults, ranging in age from 60 to 95 years. Of these 433 participants, 71 reported engaging in video game co-play with their younger family members, whereas 362 reported no co-play engagement. For analyses examining predictors of co-play behavior (H1), participants who reported no co-play were coded as 0 to indicate the absence of engagement. However, mediation analyses requiring the continuous co-play composite were restricted to participants reporting co-play (n=71). Among these co-players, 41 were female and 30 were male, and the majority were Asian (53/71, 75%), followed by White and Hispanic participants. The gender composition of the full sample was 289 female participants and 144 male participants. In terms of ethnicity, the full sample included 252 Asian participants, 96 White participants, and 85 Hispanic participants.

### Ethical Considerations

All procedures were approved by the university’s institutional review board (1655269-1). College students were recruited through a study subject pool and received course extra credit for assisting with recruitment. A link to a survey on Qualtrics was provided after each college student gave informed consent; the survey requested the email address of 1 older adult family member aged 60 years or older. The system automatically sent the survey to the electronic address provided by the college student’s family member. Older adult participation was voluntary, and no monetary or nonmonetary incentives were provided to older adult participants. The IP address was used as a filter to ensure that respondents were not located at the same physical address and to prevent multiple survey submissions from the same individual, providing supplemental verification of the reported long-distance nature of intergenerational dyads. The web-based survey was administered using a secure online platform between September 2020 and June 2021, and study procedures are reported in accordance with the CHERRIES (Checklist for Reporting Results of Internet E-Surveys) guidelines (Checklist 1). Because the survey was distributed via individualized referral emails rather than a public website, standard web-based response metrics, such as view rate and participation rate, could not be calculated.

### Measures

### Overview

The survey began with demographic information questions (ie, gender, age, and race). The rest of the survey had items measuring video game co-playing, perceived social support, and online communication attitudes. Participants were instructed to answer the items for their video game co-playing and perceived social support while considering their familial counterparts.

#### Online Communication Attitudes

This factor was measured with the OSD and OSC subscales [6]. OSD included 7 items, and OSC had 6 items. Both factors were framed as 7-point Likert-type scales (1=strongly disagree and 7=strongly agree). The OSD subscale included statements such as “I feel like I can sometimes be more personal during internet conversations” and “I feel like I can be more open when I am communicating online.” The OSC subscale contained items such as “Without the internet, my social life would be drastically different” and “I would communicate less with my friends if I could not talk with them online.” OSD (Cronbach *α*=0.91) and OSC (Cronbach *α*=0.83) for older adult participants showed good reliability.

#### Video Game Co-Play

Older adults’ engagement in collaborative video game play with younger family members was assessed using 3 items adapted from Wang et al [3]. Participants reported (1) the typical duration of a video game session with a younger family member (eg, “When you play video games with your younger family member, about how long do you play at a time?”), (2) the percentage of their total gaming time spent co-playing with that family member, and (3) the estimated percentage of the younger family member’s gaming time spent playing with them. These items captured complementary behavioral and perceptual indicators of co-play frequency and mutual engagement. Only participants who reported engaging in video game co-play completed these items. Participants who indicated no co-play engagement were not administered the follow-up items and were treated as missing for analyses requiring the continuous co-play composite. For analyses examining predictors of co-play behavior (H1), non–co-players were coded as 0 to reflect the absence of engagement. To construct the composite index, each item was standardized (*z*-score) to account for differences in scale and metric and then averaged to form a single co-play score, with higher values indicating greater engagement in intergenerational co-play. The composite demonstrated good internal consistency (Cronbach *α*=0.83).

#### Perceived Social Support

This factor was assessed using the UCLA social support inventory [53]. This scale measured informational (5 items), instrumental (5 items), and emotional support (10 items). The items were framed as a 5-point Likert-type scale (1=never to 5=very often). The items were reworded to target intergenerational relationships. Sample items included “how often did your younger/older family member provide assistance to you within the past three months?” and “how satisfied or dissatisfied have you been with the love and caring you’ve received from your younger/older family member within the past three months?” The scale showed good reliability for the older adult participants (Cronbach *α*=0.88).

## Results

The mean scores for online communication attitudes, video game co-play frequency, and perceived social support were calculated. Descriptive statistics presented in Tables1  2 reflect the full analytic sample (N=433). All regression and mediation analyses controlled for age, gender, and race (with White participants as the reference category) [54,55]. Regression analyses examining predictors of video game co-play behavior (H1) were conducted using the full sample (N=433), with participants who reported no co-play coded as 0 to indicate the absence of engagement. Hierarchical regression analyses were conducted for H2, with age, gender, and race entered in step 1 and video game co-play entered in step 2 to examine its incremental contribution to perceived social support. Mediation analyses (H3 and H4) were conducted using PROCESS Model 4 [56]. Because the continuous co-play composite was calculated only for participants reporting co-play engagement, cases with missing mediator values were excluded by PROCESS, resulting in a mediation sample of 71 participants. Indirect effects were estimated using 5000 bootstrap samples to generate 95% CIs.

**Table 1.** Descriptive statistics among study variables (N=433).

Study variables	Range	Minimum	Maximum	Mean (SD)
Online social connection	6.00	1.00	7.00	2.88 (1.29)
Online self-disclosure	5.57	1.00	6.57	2.72 (1.27)
Video game co-play score^a^^b^^c^	5.54	−0.32	5.22	−0.00 (0.86)
Perceived informational social support	4.26	1.17	5.43	3.40 (0.74)
Perceived instrumental social support	4.50	1.00	5.50	3.31 (0.80)
Perceived emotional social support	3.75	2.00	5.75	4.05 (0.67)

a The video game co-play score is a composite variable derived from 3 standardized items: typical session duration, percentage of the older adult’s gaming time spent co-playing, and percentage of the younger relative’s gaming time spent with the older adult.

b The composite was calculated among participants reporting video game co-play engagement (n=71).

c Higher scores indicate greater frequency and intensity of video game co-play.

**Table 2.** Pearson correlations among study variables (N=433).

Study variables	1	2	3	4	5	6
Online social connection	—					
Online self-disclosure	0.36^a^	—				
Video game co-play score^b^	0.09	0.19^a^	—			
Perceived informational social support	0.03	0.14^c^	0.16^a^	—		
Perceived instrumental social support	−0.10	0.09	0.21^a^	0.62^a^	—	
Perceived emotional social support	0.001	0.07	0.09	0.59^a^	0.55^a^	—

a *P*<.001.

b Regression and mediation analyses involving the continuous co-play composite were restricted to participants reporting co-play (n=71).

c *P*<.01.

H1 posits that older adults’ online communication attitudes of (a) OSC and (b) OSD would be associated with video game co-play behavior. Hierarchical regression analyses were conducted, with age, gender, and race entered at step 1 and online communication attitudes entered at step 2. For OSC, step 1 model was significant (*F*_3,429_=3.53; *P*=.03) and explaining 2% of the variance (*R*²=0.02). However, the addition of OSC in step 2 did not significantly improve the model (Δ*R*²=0.01, *F* change_1,428_=3.10; *P*=.08). The final model was significant (*F*_4,428_=3.40; *P*=.02), explaining 3% of the variance (*R*²=0.03), but OSC was not significantly associated with video game co-play behavior (*b*=0.06, SE=0.03, 95% CI −0.01 to 0.12; *P*=.08). For OSD, step 2 did significantly improve the model (Δ*R*²=.04, *F* change_1,428_=12.85; *P*<.001). The final model was significant (*F*_4,428_=6.70; *P*<.001), explaining 6% of the variance (*R*²=0.06). Moreover, OSD was significantly associated with video game co-play behavior (*b*=0.12, SE=0.03, 95% CI 0.05-0.18; *P*<.001). CIs were estimated using 5000 bootstrap samples with percentile-based intervals.

H2 posits that video game co-play would be positively associated with perceived (a) informational, (b) instrumental, and (c) emotional social support among older adults in long-distance relationships. Hierarchical regression analyses were conducted, with age, gender, and race entered in step 1 and video game co-play entered in step 2 (n=71). For informational support, the step 1 model was significant (*F*_3,67_=7.01, *R*²=0.06; *P*<.001). The addition of video game co-play in step 2 significantly improved the model (Δ*R*²=0.02, *F* change_1,66_=7.10; *P*=.009 ). The final model was significant (*F*_4,66_=6.90; *P*<.001), explaining 8% of the variance (*R*²=0.08). Video game co-play was positively associated with informational support (*b*=0.11, SE=0.04, 95% CI 0.03-0.19; *P*=.01). For instrumental support, the step 1 model accounted for 11% of the variance (*R*²=0.11, *F*_3,67_=13.15; *P*<.001). Adding video game co-play significantly increased explained variance (Δ*R*²=0.03, *F* change_1,66_=13.92; *P*<.001). The final model was significant (*F*_4,66_=13.62; *P*<.001), accounting for 14% of the variance (*R*²=0.14), and video game co-play was positively associated with instrumental support (*b*=0.16, SE=.04, 95% CI 0.08-0.24; *P*<.001). For emotional support, the step 1 model including age, gender, and race was not statistically significant (*F*_3,67_=1.65; *P*=.16), explaining 1% of the variance (*R*²=0.01). Adding video game co-play in step 2 did not significantly improve the model (Δ*R*²=0.01; *F* change_1,66_=3.76; *P*=.07). The final model was not statistically significant (*F*_4,66_=2.07; *P*=.07), explaining 2% of the variance (*R*²=0.02). Consistent with the overall model, video game co-play was not significantly associated with emotional support (*b*=0.07, SE=.04, 95% CI −0.001 to 0.15; *P*=.053).

**Figure 1.**
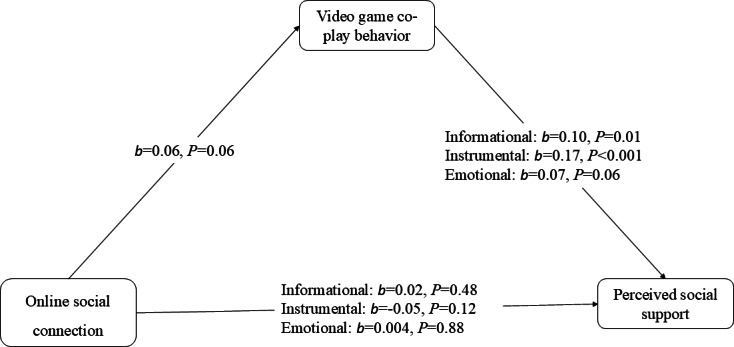
Mediation model of online social connection, video game co-play behavior, and perceived social support. This figure illustrates the mediation model examining the association between online social connection (OSC), video game co-play behavior, and perceived social support among older adults. Values represent unstandardized regression coefficients (*b*). The association between OSC and video game co-play was not statistically significant (*b*=0.06, SE=0.03; *P*=.06). Indirect effects were estimated using 5000 bootstrap samples. All paths were adjusted for age, gender, and race (White as the reference category).

H3 posits that video game co-play will mediate the relationship between OSC and (a) informational, (b) instrumental, and (c) emotional social support among older adults in long-distance family relationships. Mediation analyses (PROCESS Model 4; 5000 bootstrap samples) indicated that the model predicting video game co-play was significant (*F*_4,66_=4.18; *P*<.001), explaining 5% of the variance (*R*²=0.05). However, because no significant association was found between OSC and video game co-play (*b*=0.06, SE=0.03, 95% CI −0.001 to 0.13; *P*=.06), the mediation hypothesis was not supported (Figure 1).

H4 posits that video game co-play mediates the relationship between OSD and (a) informational, (b) instrumental, and (c) emotional social support among older adults in long-distance family relationships. For H4a, mediation analyses (PROCESS Model 4; 5000 bootstrap samples) indicated that the association between OSD and informational support was partially mediated by video game co-play. The model predicting video game co-play was significant (*F*_4,66_=5.57; *P*<.001), explaining 6% of the variance (*R*²=0.06). Higher OSD was associated with greater video game co-play (*b*=0.11, SE=0.03, 95% CI 0.04-0.17; *P*<.001). The overall model predicting informational support was also significant (*F*_5,65_=6.61; *P*<.001), accounting for 9% of the variance (*R*²=0.09). Video game co-play was positively associated with informational support (*b*=0.09, SE=0.04, 95% CI 0.01-0.17; *P*=.02). The direct effect of OSD on informational support remained significant after including video game co-play in the model (*b*=0.06, SE=0.03, 95% CI 0.001-0.11; *P*=.04), indicating partial mediation. The indirect effect of OSD on informational support through video game co-play was statistically significant (*b*=0.01, SE=0.01, 95% CI 0.001-.03).

For H4b, mediation analyses (PROCESS Model 4; 5000 bootstrap samples) supported full mediation of the association between OSD and instrumental support through video game co-play. The model predicting video game co-play was significant (*F*_4,66_=5.57; *P*<.001), explaining 6% of the variance (*R*²=.06); OSD was positively associated with co-play (*b*=0.11, SE=.03, 95% CI 0.04-0.17; *P*<.001). The overall model predicting instrumental support was significant (*F*_5,65_=11.5; *P*<.001), accounting for 14% of the variance (*R*²=0.14), and co-play was positively associated with instrumental support (*b*=0.15, SE=0.04, 95% CI 0.07-0.24; *P*<.001). The indirect effect of OSD on instrumental support through co-play was statistically significant (*b*=0.02, SE=0.01, 95% CI 0.005-0.03), while the direct effect of OSD on instrumental support was not significant after including co-play (*b*=0.03, SE=0.03, 95% CI −0.03 to 0.09; *P*=.36), consistent with full mediation.

**Figure 2.**
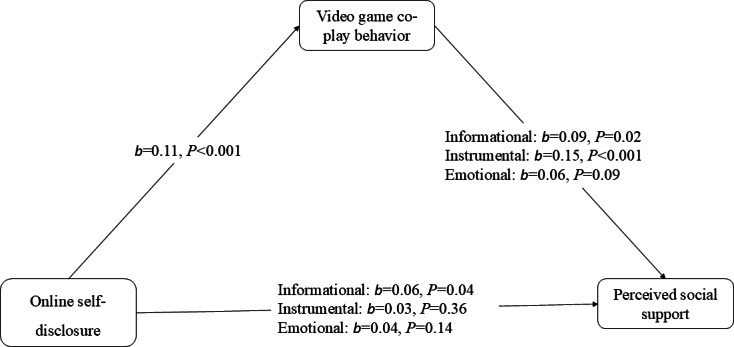
Mediation model of online self-disclosure, video game co-play behavior, and perceived social support. Mediation model examining the association between online self-disclosure (OSD) and perceived social support through video game co-play among older adults. Values represent unstandardized regression coefficients (*b*). OSD was positively associated with video game co-play (*b*=.11, SE=.03; *P*<.001). Video game co-play was positively associated with informational support (*b*=.09, SE=.04; *P*=.02) and instrumental support (*b*=.15, SE=.04; *P*<.001) but not emotional support (*b*=.06, SE=.04; *P*=.09). Indirect effects were estimated using 5000 bootstrap samples. All paths were adjusted for age, gender, and race (White as the reference category).

For H4c, mediation analyses (PROCESS Model 4; 5000 bootstrap samples) did not support mediation of the association between OSD and emotional support through video game co-play. The model predicting video game co-play was significant (*F*_4,66_=5.57; *P*<.001), explaining 6% of the variance (*R*²=0.06). However, the overall model predicting emotional support was not statistically significant (*F*_5,65_=2.11; *P*=.051), explaining 3% of the variance (*R*²=0.03). Video game co-play was not significantly associated with emotional support (*b*=0.06, SE=0.04, 95% CI −0.01 to 0.13; *P*=.09). Consistent with this pattern, the indirect effect of OSD on emotional support through co-play was not statistically significant (*b*=0.01, SE=0.01, 95% CI −0.001 to 0.02; Figure 2).

## Discussion

### Principal Findings

This study advances understanding of how older adults engage with interactive digital platforms to sustain long-distance intergenerational bonds by extending UGT into the context of aging and digitally mediated family relationships. While UGT has traditionally been applied to explain media selection and individual-level gratifications [15], these findings highlight its relevance for understanding how older adults’ online communication attitudes translate into interactive, relational digital practices. In particular, OSD emerged as a key motivational driver of video game co-play, which, in turn, was associated with enhanced informational and instrumental social support [7,39]. These findings position video game co-play not as passive media consumption but as an active, socially embedded digital health behavior through which older adults seek connection, reciprocity, and practical support within intergenerational family networks.

Although the findings indicate meaningful associations between video game co-play and perceived social support, only a small proportion of older adults in the sample reported engaging in co-play with younger family members (n=71). This limited prevalence suggests that intergenerational gaming is not yet a normative practice among older adults and may reflect broader barriers related to digital literacy, access, physical ability, or generational perceptions of gaming. The modest size of the co-player subsample necessarily limits statistical power and warrants cautious interpretation of the mediation findings, which should be viewed as exploratory rather than confirmatory. At the same time, the low uptake of co-play itself constitutes an important empirical insight, underscoring the need for targeted digital inclusion strategies that lower entry barriers and expand access to interactive digital activities for aging populations. These structural and attitudinal barriers warrant explicit consideration when interpreting these findings and when evaluating the broader applicability of video game co-play as a digitally mediated practice for aging populations.

### Barriers to Access and the Digital Divide

Although the findings suggest that video game co-play may serve as a digitally mediated pathway to informational and instrumental social support among older adults, these benefits are not equally accessible to all aging populations. Structural barriers, such as access to gaming devices, reliable internet connectivity, and appropriate software, remain persistent challenges, particularly for older adults with limited socioeconomic resources [57,58]. In addition, individual-level barriers, including limited prior gaming experience, lower digital confidence, age-related sensory or cognitive limitations, and the usability demands of commercial video games, may further constrain participation in video game co-play [39,59]. These barriers highlight the uneven distribution of opportunities to engage in interactive digital practices and underscore the importance of considering digital inequities when interpreting these findings [60-62].

Importantly, the current sample likely reflects older adults who already possess a baseline level of digital access and attitudinal readiness, which may partially explain the observed associations between online self-disclosure, co-play behavior, and perceived social support [39,58,59]. Engagement in video game co-play in this context often depended on younger family members initiating, facilitating, or sustaining participation, suggesting that intergenerational support may play a critical role in mitigating aspects of the digital divide [3,11,38]. From a digital health perspective, these findings emphasize the need for age-friendly design, intergenerational scaffolding, and community-based programs that reduce technological complexity and proactively address access barriers rather than assuming universal digital participation among older adults [62,63].

### Health Literacy and Culturally Relevant Digital Engagement

Beyond access, effective engagement with digitally mediated practices also requires adequate digital health literacy, including confidence, perceived relevance, and comfort with online interaction [57,58,64]. Although digital health literacy was not directly measured in this study, online communication attitudes function as theoretically meaningful proxies for older adults’ readiness to engage with interactive digital tools. In this study, OSD and OSC may be understood as psychosocial indicators of digital engagement readiness rather than measures of technical proficiency alone [8,39,59]. Older adults who feel comfortable sharing personal experiences online and who perceive digital platforms as socially meaningful may be better positioned to engage in interactive digital activities such as video game co-play and derive relational benefits from those interactions [16,41,51]. This interpretation aligns with broader digital health literature, emphasizing that motivation, trust, and perceived usefulness are central components of digital health literacy in later life [41,64].

Cultural context further shapes how older adults engage with digital technologies and how the benefits of such engagement are experienced [28,29,31]. The predominantly Asian composition of the co-playing subsample suggests that intergenerational digital engagement may be embedded within cultural norms, emphasizing family obligation, shared leisure, and instrumental support [30,32,65]. In such contexts, video game co-play may function less as recreational play and more as an extension of everyday family caregiving and relational maintenance [3,12,14]. These findings highlight the importance of culturally relevant digital health strategies that align with older adults’ relational values and lived experiences, rather than relying on one-size-fits-all approaches to digital literacy or engagement. Designing inclusive digital programs that recognize cultural variation in communication norms and family roles may enhance both participation and perceived benefits among diverse aging populations [58,63].

Building on these findings, the study contributes theoretically by clarifying how online communication attitudes translate into relational outcomes through specific forms of media co-engagement. Although prior research has broadly documented the role of ICT use in maintaining familial connections [2,60], these findings identify video game co-play as a distinct, immersive activity that mediates the relationship between online self-disclosure and perceived social support. In particular, co-play fully mediated the association between OSD and instrumental support, suggesting that interactive gameplay creates conditions conducive to practical assistance from younger family members. This interpretation aligns with prior evidence that older adults derive both emotional and functional value from collaborative, task-oriented forms of communication [3,27].

This study extends prior research on intergenerational video game co-play, which primarily focused on younger populations and parent-child dynamics within coresiding families [3]. By contrast, this study uniquely focuses on older adults in geographically dispersed families, incorporating a broader age range and explicitly examining long-distance intergenerational ties. Furthermore, while a previous study emphasized family closeness and satisfaction [3], this study disaggregates the social support construct into informational, instrumental, and emotional components, providing a more nuanced understanding of how specific forms of support are cultivated through co-play. In doing so, it reframes video game co-play as a digitally mediated relational practice for aging populations rather than merely a recreational activity.

Drawing on Cutrona and Suhr’s [43] social support typology, the results suggest that co-play enhances both informational and instrumental support through real-time problem-solving and skill-sharing. These findings align with prior evidence showing that active ICT engagement enhances the collaborative and cognitive capacities of older adults [17]. Within gameplay contexts, younger relatives may offer advice, guidance, or co-navigate challenges, reinforcing perceptions of competence and shared achievement among older players.

### Implications for Policy and Community-Based Digital Programs

Practically, these findings inform the design of family-based digital health programs and future intervention development. They align with aging and digital inclusion frameworks, such as World Health Organization’s Age-Friendly Digital Ecosystems and the Centers for Disease Control and Prevention’s Healthy Aging initiatives, which emphasize digital equity, intergenerational connection, and inclusive design [62,63,66]. Integrating co-play into these frameworks could inform future health technology policy. Echoing prior research on shared media use and relational bonds, this study shows that video games can support both emotional and task-oriented intergenerational support [14], especially for families separated by distance or mobility constraints. Providers may integrate co-play into care programs to reduce isolation. Structured co-play modules in home or community services could enhance engagement. Game features that encourage storytelling, collaboration, or personalization may enhance relational outcomes. Designers should prioritize emotional expressiveness and ease of use to maximize accessibility and meaningful interaction. By framing video game co-play as a relational digital health behavior rather than a recreational activity alone, this study aligns with aging-in-place and digital equity initiatives that emphasize socially meaningful technology use to reduce isolation among older adults [38,62,63].

Interestingly, although OSC was expected to predict co-play in video games, this relationship was not statistically significant. One explanation may be the difference between valuing online connections and engaging in specific behaviors, such as gaming. Older adults might appreciate digital connectivity but prefer more familiar tools (eg, email, video calls) over games, which may still seem youth-oriented [67,68]. Measurement limitations or ceiling effects may also have constrained variability in OSC scores, reducing predictive strength. Future research could further explore these dynamics, using qualitative approaches to examine older adults’ preferences and comfort levels with various digital platforms.

Notably, video game co-play was more strongly associated with informational and instrumental support than with emotional support. This pattern suggests that structured, goal-oriented digital interactions may be particularly effective for facilitating task-focused assistance, such as problem-solving, guidance, and practical coordination, rather than explicit emotional expression. Emotional support may be communicated through more direct or verbally intimate modalities, such as video calls or messaging [48,49], whereas gameplay contexts emphasize shared activity and collaboration. In addition, cultural norms surrounding emotional expression, especially within collectivist family systems, may privilege instrumental forms of care over overt emotional disclosure [31,32,69]. Thus, the nonsignificant association with emotional support does not imply an absence of affective value but rather highlights the importance of considering how different digital modalities afford distinct types of social support.

These patterns are consistent with cross-cultural research on aging, family interaction, and digital engagement, which demonstrates that cultural norms strongly shape both technology use and expectations of social support in later life [31,58,59]. Comparative studies suggest that, in collectivist cultural contexts, family-based digital activities often emphasize practical assistance, shared responsibility, and instrumental caregiving over explicit emotional disclosure [29,30]. Emotional support may be conveyed implicitly through sustained participation and shared activities rather than through overt verbal expression [31,32]. Viewed through this cross-cultural lens, the observed association between video game co-play and informational and instrumental support, rather than emotional support, aligns with broader evidence on how culturally embedded family practices shape digitally mediated intergenerational relationships.

The predominantly Asian composition of the sample provides an important sociocultural context for interpreting the findings. Cultural values emphasizing familial obligation, interdependence, and instrumental caregiving may shape both engagement in intergenerational co-play and perceptions of support derived from such interactions. In collectivist cultural contexts, shared digital activities may function not only as leisure but also as relational maintenance practices that reinforce family roles and responsibilities [65,70]. While this cultural specificity limits the generalizability of the findings to more individualistic populations, it also offers valuable insight into how culturally embedded family norms intersect with digital media use in later life. Future cross-cultural research is needed to examine how gaming practices and support dynamics vary across cultural, national, and socioeconomic contexts. Although participants were recruited through a US-based university, the national context was not independently verified, and the findings should therefore be interpreted within this institutional and sociocultural setting.

These findings offer practical implications for digital game design and public policy. Developers should design intergenerationally friendly features, such as accessible interfaces, inclusive narratives, and cooperative challenges that foster caregiving dialogue. Policymakers and health professionals can support co-play integration into aging services, including telehealth, community centers, and digital literacy programs. Aging agencies and inclusion initiatives should embed co-play into broader digital engagement strategies, with funding for training and partnerships with libraries, senior centers, and telehealth platforms to pilot intergenerational gaming as a psychosocial support tool. The findings should be interpreted as observational evidence that informs future intervention design rather than as demonstrating causal intervention effects.

As synthesized above, while video game co-play shows promise, equitable implementation requires explicit attention to persistent socioeconomic disparities, digital literacy gaps, and physical or cognitive limitations that constrain older adults’ access to interactive digital media [57]. Building on the barriers and literacy considerations discussed above, strategies such as device provision, subsidized internet access, and tailored digital skills training delivered through community-based settings are essential for inclusive adoption.

Intergenerational digital literacy programs, in which younger family members scaffold older adults’ engagement in co-play, may simultaneously support skill acquisition and relationship building [71]. To ensure cultural relevance, interventions should incorporate design elements aligned with diverse familial values and communication norms. Multilingual interfaces, culturally familiar imagery, and collaborative mechanics suited to collectivist traditions can enhance engagement.

Age-inclusive policies must move beyond usability to support emotionally resonant, culturally congruent experiences. This study offers empirical grounding for future interventions that design and evaluate digital tools promoting relational well-being. Emphasizing co-play as a vehicle for relational support underscores the need for culturally and socially responsive technology–enabled aging innovation.

### Limitations and Future Directions

Several limitations warrant consideration when interpreting this study’s findings. First, the study used a cross-sectional design, which limits causal inference regarding the relationships among online communication attitudes, video game co-play, and perceived social support. Although mediation analyses were theoretically grounded, the results should be interpreted as exploratory and hypothesis-generating rather than as evidence of causal mechanisms. Longitudinal or experimental designs are needed to establish temporal ordering and causal direction.

Second, the sampling strategy relied on snowball recruitment facilitated by undergraduate students, which may limit representativeness. While this approach enabled access to geographically dispersed, long-distance intergenerational dyads, a key focus of the study, it may also have introduced selection bias toward families with stronger intergenerational ties or greater digital access. In addition, the sample was predominantly composed of Asian older adults. This ethnic composition likely reflects both institutional recruitment context and cultural norms emphasizing familial obligation and interdependence. Although this provides valuable insight into culturally embedded patterns of intergenerational digital engagement and contributes to theory-building in collectivist family contexts, it constrains the generalizability of the findings to other ethnic or cultural groups. This cultural context likely shapes both the forms of support emphasized and the meanings ascribed to co-play and thus should be understood as an interpretive lens rather than a confounding artifact.

Third, only a small proportion of participants (n=71) reported engaging in video game co-play with younger family members. This limited subsample reduces statistical power and increases the risk of type 2 error, particularly in mediation analyses. As such, null findings, especially regarding emotional support, should be interpreted cautiously. At the same time, the low prevalence of co-play itself represents a meaningful empirical finding, highlighting potential barriers related to digital literacy, access, generational norms, or physical ability that warrant further investigation.

Fourth, measures of video game co-play were based on self-reported estimates of shared gaming time and involvement, which may be subject to recall bias or social desirability effects. This concern may be especially salient among older adults with varying levels of cognitive load or digital familiarity. Future research should consider incorporating objective or behavioral measures, such as gameplay logs, platform-generated usage data, or ecological momentary assessment, to enhance measurement precision.

Fifth, the long-distance nature of intergenerational dyads was based on participant reports and supplemented by IP address filtering to ensure respondents were not located at the same physical address. However, geographic distance was not independently verified, and perceived distance may differ from objective distance. Future studies may benefit from more precise geographic measures to examine how physical separation interacts with digital engagement and support dynamics. Perceived geographic distance may differ from objective distance, and this distinction may influence how intergenerational support is experienced and interpreted, as perceived separation may be more salient for relational dynamics than physical distance alone.

Finally, the study did not directly assess socioeconomic status, national context, or broader structural factors that may shape access to digital technologies and gaming practices. Economic resources, educational background, and cultural exposure to gaming may act as unmeasured confounding variables influencing both co-play behavior and perceived social support. Future research should incorporate more diverse, cross-national samples and explicitly measure socioeconomic and structural determinants of digital engagement in later life.

### Conclusion

In conclusion, this study highlights the potential of video game co-play as a meaningful, home-based, digitally mediated relational practice through which older adults may sustain intergenerational relationships across geographic distance. Rather than positioning co-play as an established intervention, the findings suggest that older adults’ online communication attitudes, particularly online self-disclosure, are associated with engagement in interactive digital activities, which, in turn, relate to perceptions of informational and instrumental social support. These patterns are consistent with UGT and extend its application to aging populations by illustrating how motivational dispositions translate into relational digital practices in later life. By situating video game co-play within culturally embedded family contexts and acknowledging structural and access-related constraints, this study underscores the importance of age-inclusive, culturally responsive digital engagement strategies for addressing social isolation among older adults.

## Supplementary material

10.2196/80541 Checklist 1 CHERRIES checklist.

## Abbreviations

CHERRIES: Checklist for Reporting Results of Internet E-Surveys
ICT: information and communication technology
OSC: online social connection
OSD: online self-disclosure
UGT: uses and gratifications theory
