# Supplementary material for: Online Communication Attitudes and Video Game Co-Play in Older Adults: Cross-Sectional Mediation Study
Source: JMIR Aging. 2026 Apr 7;9:e80541. doi: 10.2196/80541 (PMC13055934; doi:10.2196/80541)
Supplement: Checklist 1 [file aging-v9-e80541-s001.docx]

**CHERRIES Checklist**

**Checklist for Reporting Results of Internet E-Surveys (CHERRIES)**

**Design**

**Survey design**
This study employed a cross-sectional online survey design. Participants were older adults aged ≥60 years recruited through a snowball sampling approach facilitated by undergraduate students. The sample represents a non-probability convenience sample.

**IRB Approval and Informed Consent**

**IRB approval**
All procedures were approved by the university’s Institutional Review Board (Protocol #1655269-1).

**Informed consent**
Participants provided informed consent electronically prior to beginning the survey. The consent form described the purpose of the study, procedures, the voluntary nature of participation, and data confidentiality.

**Data protection**
No sensitive personal identifiers were collected. Data were collected via Qualtrics and stored securely with access restricted to the research team.

**Development and Pre-testing**

**Development and testing**
The survey was developed using validated scales from prior literature. The questionnaire was pilot-tested to ensure clarity, usability, and appropriate survey duration.

**Recruitment Process and Sample Description**

**Open vs closed survey**
This was a closed survey. Only participants invited via email (through undergraduate students) could access the survey.

**Contact mode**
Initial contact was made offline (via undergraduate students), followed by email invitations sent to older adult participants.

**Advertising the survey**
The survey was not publicly advertised. Recruitment occurred through personal referral by undergraduate students to eligible older adult family members.

**Survey Administration**

**Web/E-mail**
The survey was administered online via Qualtrics, and responses were automatically recorded.

**Context**
Participants accessed the survey through individualized email invitations, minimizing external contextual influences.

**Mandatory/voluntary**
Participation was voluntary.

**Incentives**
No incentives were provided to older adult participants. Undergraduate recruiters received course credit.

**Time/Date**
Data were collected between September 2020 and June 2021.

**Randomization of items**
Survey items were not randomized.

**Adaptive questioning**
Adaptive questioning was used (e.g., only participants reporting video game play received follow-up co-play items).

**Number of items**
The survey included demographic items and validated scale items assessing online communication attitudes, co-play, and social support.

**Number of screens/pages**
The survey was distributed across multiple pages in Qualtrics.

**Completeness check**
Participants could skip questions; no forced responses were implemented. Data completeness was assessed post hoc.

**Review step**
Participants were able to review and change responses prior to submission.

**Response Rates**

**Unique site visitor**
Not applicable, as the survey was distributed via individualized email invitations rather than a public website.

**View rate**
Not available due to the closed, email-based distribution method.

**Participation rate**
Not available due to the recruitment design.

**Completion rate**
Completion rate was not calculated due to the closed, email-based survey design; however, incomplete and low-quality responses were excluded during data cleaning.

**Preventing Multiple Entries**

**Cookies used**
Cookies were not used.

**IP check**
IP addresses were used to ensure that respondents were not located at the same physical address and to reduce the likelihood of duplicate entries.

**Log file analysis**
No additional log file analyses were conducted.

**Registration**
No formal registration system was used.

**Analysis**

**Handling of incomplete questionnaires**
Participants with excessive missing data or incomplete responses were excluded. Additionally, participants with missing co-play data were excluded where appropriate.

**Atypical timestamps**
Responses completed in less than 300 seconds were excluded based on pilot testing benchmarks.

**Statistical correction**
No weighting or statistical corrections were applied. Analyses were conducted using regression and mediation models with bootstrapping.
